# Association of tooth loss and nutritional status in adults: an overview of systematic reviews

**DOI:** 10.1186/s12903-024-04602-1

**Published:** 2024-07-24

**Authors:** Pragati Kaurani, Pradnya Kakodkar, Anamitra Bhowmick, Rupandeep Kaur Samra, Vineeta Bansal

**Affiliations:** 1 Department of Prosthodontics and Crown & Bridge, Mahatma Gandhi Dental College and Hospital, Jaipur, India; 2 KT Consultancy, Pune, India; 3 West Bengal University of Health Sciences, Kolkata, West Bengal India; 4 Department of Prosthodontics and Crown & Bridge, DJ College of Dental Sciences and Research, Modinagar, Ghaziabad, Uttar Pradesh 201204 India; 5 Department of Clinical Nutrition and Dietetics, Mahatma Gandhi Medical College and Hospital, Jaipur, India; 6 House no.10, Doctor’s Colony, Bhadson Road, Patiala, Punjab 147001 India

**Keywords:** Nutritional status, Edentulous, Elderly, Tooth loss, Number of teeth

## Abstract

**Background:**

Association of tooth loss and nutritional status has been widely researched with conflicting results. This overview aimed to analyse and summarize findings from systematic reviews on association of tooth loss with nutritional status, in view of their quality assessment and methodological characteristics.

**Methods:**

Overview was conducted as per Cochrane Overviews of Reviews guidelines. 5 databases (PubMed, Dentistry and Oral Sciences Source, Scopus, Cochrane Register of Systematic Reviews, Epistemonikos.org) and one online source (Google Scholar) were searched for systematic reviews published between 2010 - July 2022, with inclusion criteria; population: participants aged 18 years or above, intervention/exposure: loss of teeth, comparison: not applicable, outcome: nutritional status, study: systematic reviews and meta-analysis. Reviews on association of prosthetic interventions with nutritional status were not included. Data were extracted for study characteristics, details of primary studies, and main findings. Narrative synthesis of data, overlap of primary studies and quality assessment of studies were done using AMSTAR 2 tool (A Measurement Tool to Assess Systematic Reviews).

**Result:**

Of 1525 articles found, seven systematic reviews were selected (four were systematic reviews, three were systematic reviews with meta-analysis). Five studies showed some or positive association, one found weak association and for one study the association was unclear. Overlap of primary studies was ‘very slight’. Meta-analysis of two studies concluded fully or partially edentulous individuals were more likely to be malnourished or at risk of malnutrition, (RR = 1.095, 95% CI 1.007 to 1.190, *p* = 0.033. RR = 1.22; 95% CI 1.11 to 1.32 *p* < 0.01), but one found that edentulism was not associated with malnutrition. (RR = 1.072, 95% CI 0.9657-1.200). Quality assessment revealed four studies were ‘high’, one was ‘low’ and two were ‘critically low.’

**Conclusion:**

This overview confirms the association between tooth loss and nutritional status specially in elderly. It is evident that tooth loss increases the likelihood of poor nutritional status. Overall, studies show high heterogeneity in the methodology and quality assessment reveals low confidence in the available evidence. Future studies should use standard assessment tools for tooth loss and nutritional status.

**Supplementary Information:**

The online version contains supplementary material available at 10.1186/s12903-024-04602-1.

## Introduction

Teeth form an integral part of the oral cavity, impacting an individual’s general health. A functional and healthy dentition is an essential aspect of oral health; thus, tooth loss is considered an indicator of poor oral health [1, 2]. As global life expectancy and the elderly population increase, the risk of tooth loss is anticipated to increase. Consequently, tooth loss is regarded as one of the most significant oral public health issues worldwide [3–5].

The major etiological factors for the loss of teeth are periodontal conditions, dental caries, trauma, and orthodontic extractions [6–8]. Apart from these, several other factors associated with tooth loss are age, oral health behaviours, availability of dental services, and socio-behavioural factors [9]. With the loss of teeth, various degrees of oral disabilities are primarily known to occur that can affect adults such as oral frailty (less than 20 teeth) and oral hypofunction [10–12].

The temporal sequence of tooth loss leading to reduced nutritional intake has been studied extensively [13–18]. Loss of teeth causes functional impairment and chewing disabilities, affecting the intake of nutritious food and reducing the pleasure of eating food [13–15]. Moreover, individuals with missing teeth are known to change or adapt their food preferences and swallow coarser particles or take considerable bites to compensate for poor mastication [18]. Such adaptations may lead to imbalances in the diet or gastrointestinal disturbances, leading to nutritional deficiencies and affecting the overall general health [18].

Several studies have been undertaken, and varied results have been reported to substantiate the evidence of the association of tooth loss with nutritional intake [19–23]. Over the years, several reviews were published to systematically synthesize the available evidence from primary studies [13, 16, 17, 24–28]. This available wealth of reviews necessitates the synthesizing and evaluating all the available evidence in the form of an overview to enable a clearer understanding of the association [29, 30]. Thus, the current overview aimed to summarize findings of systematic reviews undertaken to study the effect of loss of teeth on nutritional status in adults and further critically evaluate the quality of these systematic reviews and their methodological characteristics. The focused research question of this overview was: What is the association of tooth loss with nutritional status in adults?

## Methodology

### Protocol registration

The protocol was prepared as per the guidelines of Cochrane Overviews [31]. A modified version of the Preferred Reporting Items for Overviews of Reviews (PRIOR) statement was used for reporting this overview [32]. The prior protocol was registered in the PROSPERO database (CRD42021284395). To have a broader understanding of tooth loss, post-hoc amendment in the protocol for the inclusion criteria was done (as agreed by all authors) for the term loss of teeth to include studies on tooth loss as a component of oral health and function.

### Inclusion and exclusion criteria

Inclusion criteria were as follows, Population: No restriction on the type of participants was kept, that included participants aged 18 **years** or above, irrespective of recruitment setting, country and health status. Intervention or Exposure observed: Loss of teeth (partial or complete edentulism) or number of teeth present reported either through self-reports or using a clinical examination. Comparison: not applicable. Outcome: Nutritional status was defined as “a physiological state of an individual, which results from the relationship between nutrient intake and requirements, and from the body’s ability to digest, absorb and use these nutrients [33].” With an expanded understanding of nutritional status, food or dietary intake, malnutrition or being at risk of malnutrition as outcome measures were included. Study Design: Published systematic reviews or meta-analysis (SRs/MAs) using the understanding of systematic reviews (SRs) as given by Martinic et al. were considered [34]. Supplementary primary studies were not included. Further, SRs/MAs that provided insufficient or unclear for measuring tooth loss or nutritional status were excluded.

Search strategy and searches: Three bibliographic electronic databases, Medline via PubMed, Dentistry and Oral Sciences Source (DOSS) via EBSCOhost, Scopus; two SRs databases, Cochrane Register of Systematic Reviews, epistemonikos.org and one online source, Google Scholar were searched for English peer-reviewed SRs/MAs published between 2010 until 30th July 2022. Studies published prior to 2010 were not considered as they may not reflect current understanding of SRs/MAs, (such as searching in more than one database) [34]. Manual citation searching of the reference lists of retrieved articles was done and one online protocol registry PROSPERO was searched for potential articles. Key terms “nutritional status”, “nutrition”, “nutrition assessment”, “edentulism” and “tooth loss” and the search filter ‘systematic review’ were used. Supplementary File Table 1 depicts the search strategy used.

**Table 1:** Characteristics of the included systematic reviews/meta-analyses (SR/MAs) in the study

Sr. No.	Author name and year of publication	Study design	Aim or Research Question stated.	PICO	Type of studies included	Limitations reported in the SR	Relevant Overall conclusion
1	AlgraY [24]. 2021.	SR	To examine the association between malnutrition and oral health in older individuals (≥ 60 years of age).	NS	Observational and interventional studies	Statistical and clinical heterogeneity observed in the included studies. Cross-sectional design of the included studies.	Malnutrition is related to the conditions of the hard and soft tissues of the mouth.
2	Gaewkhiew P [25] 2017	SR	To systematically review longitudinal evidence on how tooth loss affects dietary intake and nutritional status among adults.	P = adults aged 18 years or above, I = Tooth loss measured at least once during the duration of the study (baseline assessment) through self-reports or clinical examination. O = Dietary/food/ nutrient intake.	Longitudinal/Panel studies.	High variability in methods used to measure exposures and outcomes. Search limited to three electronic data bases and did not fully search unpublished studies.	There is weak evidence on the association of tooth loss on nutrition and diet.
3	Hussain S. [26] 2020.	SR and MA	To determine how poor oral health can affect the nutritional status of older adults.	P = Greater than 65 years, I = oral health outcomes, C = none, O = MNA, MNA-SF	Cohort and Cross sectional studies.	Only studies with patients over and 65 years were included. Significant variability in the measurement of oral health variables.	Edentulism, and chewing problems was associated with high risk of malnutrition. RR = 1.095, 95% CI 1.007 to 1.190, *p* = 0.033.
4	Lancker V.A. [28] 2012.	SR	To determine the association between oral health status and malnutrition in elderly residing in a long-term care facility.	P = Elderly patients living in long-term care facilities (mean age range->/=60 years-89.6 years), I = oral health status and malnutrition, C = NS, O = Causal relationship between three groups of oral health problems and malnutrition.	Cross sectional study.	Largely, the quality of the included studies was medium. Absence of prospective study, non-probability sampling techniques designs, managing outliers were reported methodological limitations.	Oral health status and malnutrition are independently associated in patients residing in long -term facility.
5	Tada A. [16] 2014.	SR	To systematically review the published findings on the association of mastication and mastication associated factors with food and/or nutrient intake in the independent elderly of community dwelling.	P = Subjects aged 50 years or older, or population with a mean age > 55 years, living independently O = Self-reported or interviewed food intake. Nutrient intake calculated from food intake Nutrient profile measured on serum and blood Analysis = Any association between oral health and food and/or nutrient intake.	Cross-sectional studies.	NS	Most of the cross-sectional studies demonstrated the association of mastication with food and /or nutrient intake with differential impact on elderly.
6	Toniazzo M. [13] 2017	SR and MA	The review aimed to evaluate and compare the oral health status older adults with normal nutrition, at risk of malnutrition and malnourished individuals.	P = Subjects at least 60 years old; I = tooth loss or number of teeth present, edentulous, Decayed, Missing or Filled Index (DMF), functional teeth units (FTU). O = nutritional evaluation, such as MNA or its shortform or SGA.	Observational and interventional studies.	High variability in the studies was observed. Subrogated oral health outcomes were analysed.	Functional Teeth Units and number of teeth were significantly associated with nutritional status. Edentulism was not associated with nutritional status. (RR = 1.072, 95% CI 0.9657-1.200)
7	Zelig R [17] 2020.	SR and MA	In older adults (≥ 60 y of age and living in developed countries), what are the associations between tooth loss (< 28 teeth) and nutritional status as assessed by a validated nutrition screening/assessment tool ?	P = older adults (≥ 60 y of age and living in developed countries). I = with tooth loss (< 28 teeth) or tooth replacement (removable full or partial dentures, implants, and implant supported dentures. O = nutritional status as assessed by a validated nutrition screening/assessment tool. S = Randomized Control trials, Case-Control studies, cross-sectional and cohort studies.	Randomized controlled trials or cohort, case-control, cross-sectional, or interventional studies	Use of observational data. Limitation in the ability to address impact on mastication due to lack of consensus on measuring tooth loss.	Edentulous individuals or who lacked functional dentition had 21% increased chances of being at risk of malnourished or being malnourished. (RR 1.21; 95% CI, 1.11 to 1.32; I 2 = 70%).

NS = Not stated, SR = Systematic Review, MA = Meta-analysis. P = Population, I = Intervention, C = Control, O = Outcome measure. MNA = Mini nutrition assessment, MNA-SF = Mini nutritional assessment short form, SGA = Subjective global assessment. RR = Risk ratio

### Screening

Using the developed search strategies, studies were searched in different databases and exported to Rayyan Software, after which de-duplication was performed. Titles and abstracts were screened using the inclusion and exclusion criteria by two reviewers (PK and AB) individually with a good agreement (Kappa Statistics 0.92). Full texts of the articles were downloaded and further independently verified for the eligibility by the same two reviewers. Wherever there was a dispute, the third reviewer (PK2) was referred and the dispute was resolved. The requirement to contact the author/co-authors of the SRs for clarification was not felt.

Citation matrix was generated by one reviewer (AB) and subsequently checked for accuracy by a second reviewer (PK) [35]. To calculate the degree of overlap of the included primary studies, the Corrected Coverage Area was calculated [35]. The decision tool by Pollock et al. was used to decide on the inclusion of overlapping SR [36].

### Data extraction

Data items were extracted by two reviewers individually (PK and AB) and verified by a third reviewer (PK2). The data were grouped using the following Study characteristics: author, year of publication and study design, PICOT, information of sources used, duration of search. Details of the primary studies included in the SR: language, study designs and country. Main findings for the data analysis: measures of tooth loss, measures of nutritional status, methods and results of assessing risks of bias and quality of the primary studies, a summary of MA and overall conclusion of the SR. Discrepant data was searched. Missing data was mentioned as ‘not stated’ or NS.

### Risk of Bias and quality assessment

The overall quality assessment of the included SRs was performed independently by two reviewers (PK and RKS) and using AMSTAR 2 (A Measurement Tool to Assess Systematic Reviews) tool, and conflicts were resolved by the third reviewer (PK2) [37]. To summarize risk of bias assessments (RoB) and quality assessments of primary studies of individual systematic review, data was directly taken from included SRs (rather than assessing the risk of bias anew).

Data of interest were presented using narrative summary synthesis and supported using text, figures and tables.

## Results

### Search findings

**Fig. 1:**
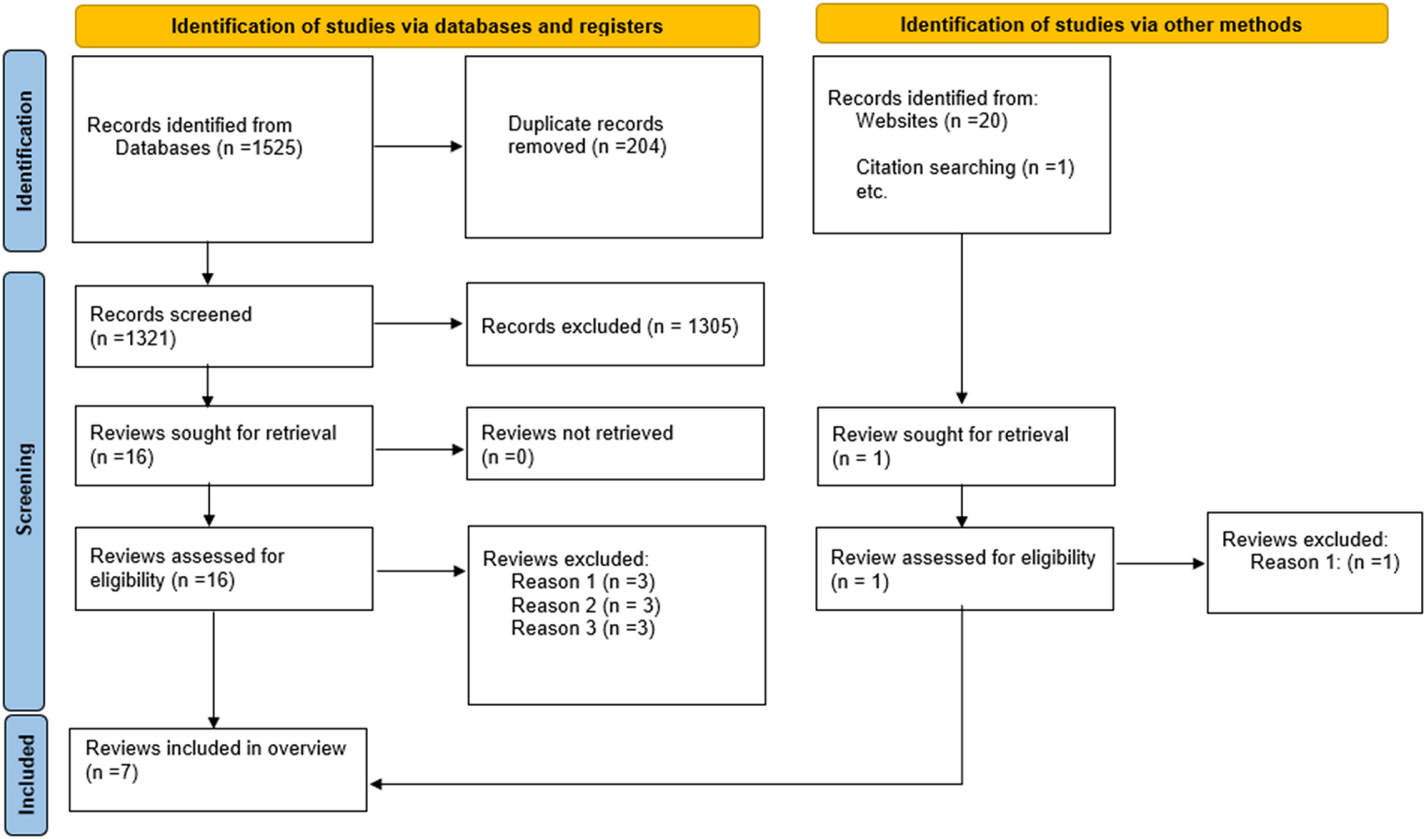
PRISMA 2020 flow diagram, including searches of databases, registers and other sources Reasons for Exclusion: Reason 1: Review done before 2010, Reason 2: Review did not classify as systematic reviews, Reason 3: Review did not mention methods used to assess tooth loss or nutritional status clearly

The electronic search resulted in 1525 articles, and the de-duplication eliminated 204 articles. The initial round of screening resulted in elimination of 1305 articles. Full texts for the remaining 16 studies were retrieved and further evaluated, which resulted in exclusion of 9 studies due to the following reasons: done before 2010 [38–40], review did not classify as systematic review [27, 41, 42], unclear methods to assess tooth loss or nutritional status [43–45]. Finally, seven SRs/MAs were selected for inclusion [13, 16, 17, 24–26, 28]. The methodology adopted for the search and selection process is depicted in the PRISMA 2020 flowchart. (Fig. 1)

**Fig. 2:**
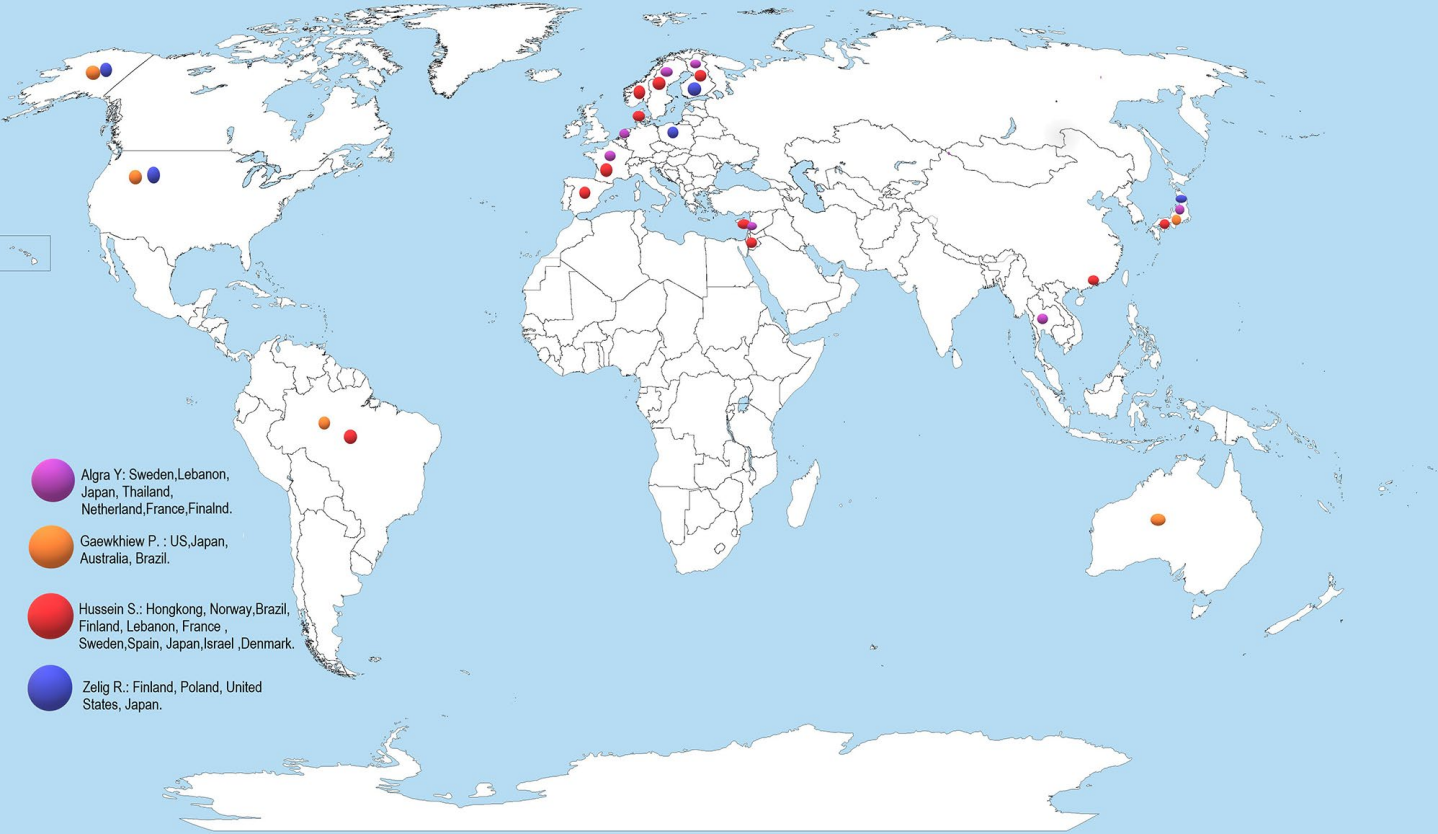
Countries where the primary studies were conducted

Study characteristics: Of the seven included SRs/MAs, four were SR [16, 24, 25, 28], and three studies were SR with MA [13, 17, 26]. Figure 2 displays the countries where primary studies were conducted. From the primary studies, no discrepant data were identified. The studies were published between the years ranging from 2011 to 2023. Most of the SRs included a population of varied population settings and age as 50 years and above, only one SR included a younger age group of 30 to 65 years [25]. Table 1 shows details of the characteristics and Table 2 shows methodology adopted in the included SRs/MAs.

**Table 2:** Details of methodology used in the included systematic reviews

Sr. No.	Author name	Information sources: Databases and grey literature.	Duration of the search	Citation searching	Protocol registration.	Was search strategy provided?	Applied limitations	Independent study selection process	Reasons provided for exclusion	Languages of studies included	Conflict of Interest	Funding details provided
1	Algra Y. et al. [ 24]	PubMed, Embase (Ovid), CINAHL (EBSCO), and Dentistry and Oral Sciences Source (DOSS). NS	January 2000 to May 2020	Yes	NS	No	NS	Yes	Yes	English and Dutch	None	Yes
2	Gaewkhiew P [25].	MEDLINE via PubMed, EMBASE via Ovid and LILACS via BIREME. Open Grey repository, Google Scholar and searching the internet	Up to March 2017	Yes	Yes	Yes	Yes	Yes	Yes	No language restriction.	None	Yes
3	Hussain S. et al. [26]	Medline, Web of Science, Cochrane, Embase. NS	February 2018 updated in June 2020	Yes	NS	Yes	Yes	Yes	Yes	English	None	Yes
4	Lancker V.A et al. [28]	Medline, Cinahl and Library of the Cochrane Collaboration. NS	January 1985 to May 2011.	Yes	NS	Yes	NS	Yes	Yes	English, Dutch FrenchGerman	None	None
5	Tada A. et al. [16]	PubMed, Web of Science, CochraneLibrary, and Scirus. NS	1991 and 2013	Yes	NS	Yes, key words provided.	NS	Yes	NS	English	None	Yes
6	Toniazzo M. et al. [13]	PubMed, Scopus and Embase NS	No restriction on date	Yes	NS	Yes	NS	Yes	Yes	No language restriction.	None	Yes
7	Zelig R et al. [17]	PubMed, Scopus, CINAHL, Web of Science, and MEDLINE. The Open Grey database.	January 2009 and December2019.	Done	NS	Yes	NS	Yes	Yes	English	None	Yes

Exposure and outcome measure: Both exposure and the outcome of interest had varied measures across studies. Supplementary File Tables 2 and 3 shows the different measures used for the assessment of the tooth loss and nutritional status respectively. Mini Nutritional Assessment (MNA) tool was found to be the most widely used method of assessment of nutritional status and “teeth present” was the most common measure used for tooth loss exposure.

**Table 3:** Scoring as per AMSTAR 2 tool of all the included SR/MAs in the study

Sr. No.	Authors	1	2	3	4	5	6	7	8	9	10	11	12	13	14	15	Overall Confidence in the results
1.	Algra Y et al. [24]	Yes	Partial Yes	Yes	Partial Yes	Yes	Yes	No	Yes	Yes	Yes	-		Yes	Yes	Yes	Low
2.	Gaewkhiew P. et al. [25]	Yes	Yes	Yes	Yes	Yes	Yes	Yes	Yes	Yes	Yes	-		Yes	Yes	Yes	High
3.	Hussein S. et al. [26]	Yes	No	Yes	Partial Yes	Yes	Yes	Yes	Yes	Yes	Yes	Yes	Yes	Yes	Yes	No	Critically Low
4.	Lancker V A. et al. [28]	Yes	Partial Yes	Yes	Partial Yes	Yes	Yes	Yes	Yes	Yes	Yes			Yes	No	No	High
5.	Tada A. et al. [16]	Yes	Partial Yes	Yes	Partial Yes	Yes	Yes	No	Yes	No	Yes			No	Yes	-	Critically Low
6.	Toniazzio P.M. et al. [13]	Yes	Partial Yes	Yes	Partial Yes	Yes	Yes	Yes	Yes	Yes	Yes	Yes	Yes	Yes	Yes	Yes	High
7.	Zelig R. et al. [17]	Yes	Partial Yes	Yes	Partial Yes	Yes	Yes	Yes	Yes	Yes	Yes	Yes	Yes	Yes	Yes	No	High

1: Did the research questions and inclusion criteria for the review include the components of PICO? 2: Protocol registered before commencement of the review. 3: Did the review authors explain their selection of the study designs for inclusion in the review? 4: Adequacy of the literature search. 5: Did the review authors perform study selection in duplicate? 6: Did the review authors perform data extraction in duplicate? 7: Justification for excluding individual studies. 8: Did the review authors describe the included studies in adequate detail? Item 9: Risk of bias from individual studies being included in the review. 10: Did the review authors report on the sources of funding for the studies included in the review? 11: Appropriateness of meta-analytical methods. 12: If meta-analysis was performed did the review authors assess the potential impact.13: Consideration of risk of bias when interpreting the results of the review.14: Did the review authors provide a satisfactory explanation for, and discussion of, any heterogeneity observed in the results of the review? 15: Assessment of presence and likely impact of publication bias

Number of teeth lost and nutritional status: Toniazzo and others found that mean number of teeth and FTU (Functional teeth units) were significantly associated with nutritional status, however, they were unable to make a clinical relevance to this finding. Further, they concluded that subjects who were malnourished/at risk of malnutrition had significantly fewer teeth (-0.141, 95% CI 0.278 to 0.00502) [13]. On the other hand, another study could not demonstrate a positive association of masticatory-associated factors such as teeth number and dentition status with nutritional status [16]. They concluded that factors other than masticatory factors play a role in determining the nutritional status. Algra at al studied tooth loss as a component of oral health and found extensive inter-relation between oral health and malnutrition [24]. In another study, association between dental conditions and malnutrition was evaluated in seven primary studies and all seven studies found a significant association with malnutrition [24]. Tada and others could not demonstrate a correlation and concluded that other factors than mastication were associated with food and or nutrient intake [16].

Effect of loss of teeth on dietary intake: Only one study analysed the effect of tooth loss and nutritional intake, concluding that there is poor evidence on the effect of tooth loss on dietary intake and nutritional status [25]. The study could find consistent results with only dietary cholesterol, where it was found that with loss of teeth there is reduced intake of dietary cholesterol [25].

Complete or partial edentulism as a risk factor for being malnourished: Hussein et al. concluded that partially or fully edentulous patients had 9.5% higher chances of being at risk of malnourished and older adults with chewing problems were at twice the risk of malnutrition. (RR = 1.095, 95% CI 1.007 to 1.190, *p* = 0.033) [26]. Zelig et al. reported that completely edentulous individuals or those who lacked functional dentition had a 21% increased likelihood of being at risk of malnutrition or malnourished, although they observed high heterogeneity among studies (I2 = 70%, *P* < 0.01) [17] Tonniazzo P et al. found that the relative risk for edentulism was 1.072 (95% CI 0.9657e1.200), and was not significantly different in institutionalized and noninstitutionalized individuals [13].

### Overlap and quality assessments

**Fig. 3:**
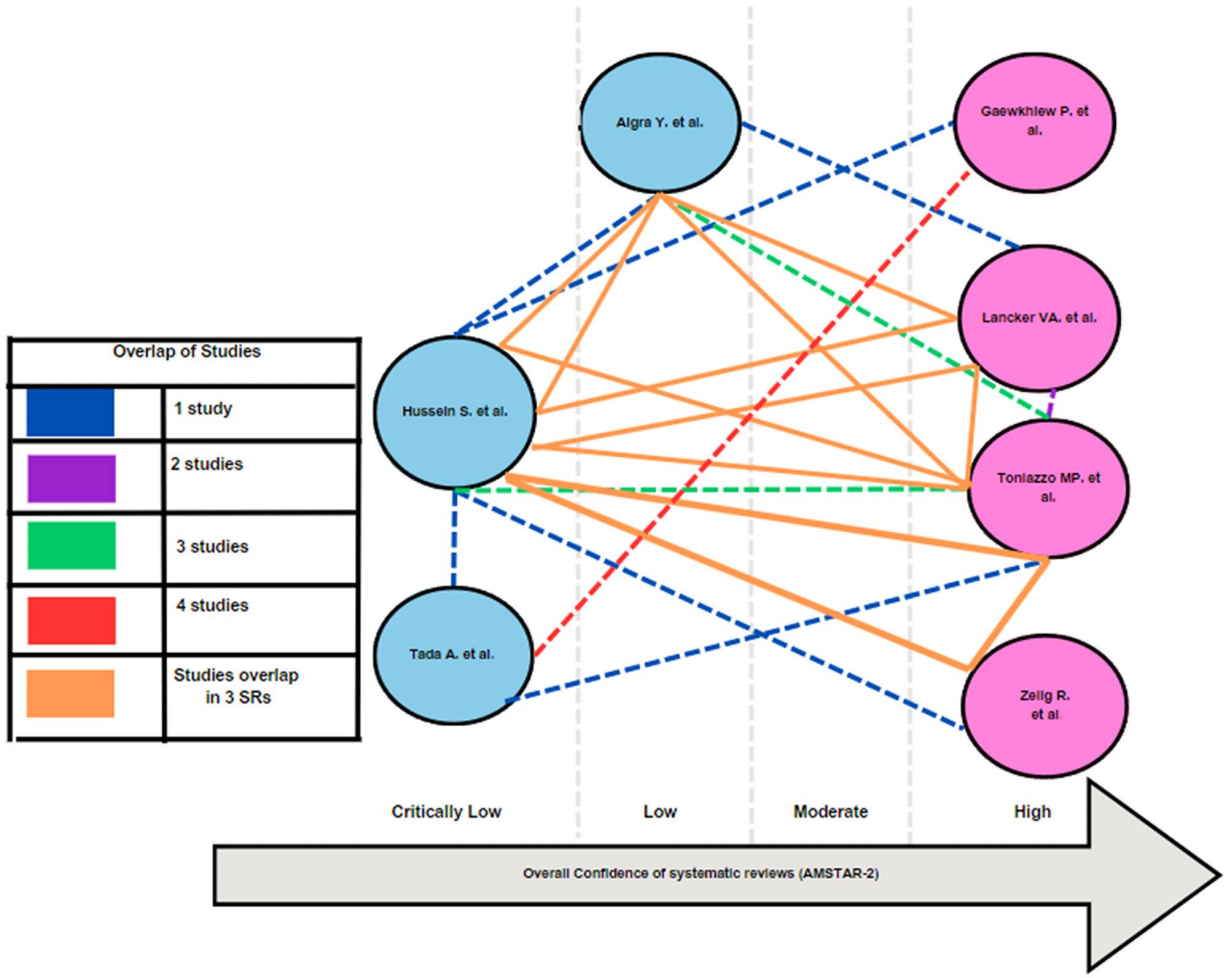
Quality of the included SR/MAs as per the AMSTAR 2 score and the overlap of studies

The overlap was calculated to be 0.04 and interpreted as ‘slight’ and thus, none of the SRs were eliminated. Citation matrix and calculation of corrected covered area are shown in Supplementary File Table 4. The results of the AMSTAR 2 tool revealed that the overall confidence in four studies was rated ‘high’ [13, 17, 25, 28], one study was rated ‘low’ [24] and in two studies was rated ‘critically low” [16, 26]. The details of the scoring of the AMSTAR 2 tool of the included studies and the overlap of the studies are depicted in Tables 3 and Fig. 3. Analysis of quality assessment done by individual studies revealed that almost all included SRs reported quality of evidence from the primary studies as ‘poor’. (Supplementary File Table 5) All studies used valid tools for RoB, while the use of Grading of Recommendations Assessment Development and Evaluation (GRADE) approach was observed in two SRs only [17, 26].

## Discussion

Given that there has been a growing body of literature that has analyzed the association between tooth loss and nutritional status, an overview became logically essential. The results indicate that tooth loss is associated with nutritional status in adults, although the currently available supporting evidence is not free from methodological inconsistencies.

Variations in study parameters can affect the results of the studies. Vast variations in sociodemographic characteristics of population and assessments of both exposure and outcome have been noted across the reviews. Furthermore, variations were observed in population settings in the studies. Toniazzo and others found that oral health has lesser influence on malnutrition in institutionalized individuals [13]. This can be attributed to the fact that institutionalized individuals may have other severe chronic diseases and polypharmacy that contribute to malnutrition [46]. On the other hand, two studies restricted their population setting in their inclusion criteria with studies based on long-term facility and individuals living independently [16, 28]. Variability in population settings across the SRs raises concerns regarding the potential effects on generalizability of the obtained results. Another variable observed was the age of included subjects, where the majority of the studies included elderly subjects with varied understanding of the included age, and only one study included younger adults (30 to 65 plus years), leading to broad age differences in the included studies [25]. These broad age variations could be due to the differences in the age categorization of elderly people in different countries.

Additionally, the causative factors associated with tooth loss and malnutrition differ in developed and emerging nations [47–50]. It was observed that most primary studies were from developed nations, with the review by Zelig et al. that included only the primary studies based in developed countries [17]. To ensure high external validity of the results, it is essential to have studies in alternative settings as differences in culture and diet can influence the results.

Another crucial aspect to consider when inferring conclusions from the current study’s findings is the potential effects of underlying systemic conditions. Four of the included SRs mentioned the adjustment for systemic conditions, Zelig et al. performed a subgroup meta-analysis and found that the risk of being malnourished is increased by 37% if medical history is adjusted for [17], two SRs excluded studies where participants had systemic conditions like or malignancies, terminal illness, dysphagia or chewing problems, musculoskeletal among others [16, 24], and one study selected studies irrespective of the health status i.e. generally healthy or with one or more morbidities [25]. Even if there is adequate nutrient intake, altered nutritional needs with underlying systematic conditions can affect the nutritional status [51]. Thus, future studies must consider the effect of underlying systemic conditions when analyzing the association.

Notably, high variability in the measures of tooth loss and nutritional status across the studies was observed. Although it is usual for health-related outcomes and exposures in observational studies of etiology to be stated, defined and measured in various ways, careful assessment is required for comparability [52]. To address the research question better, the current overview had a broader understanding of tooth loss, and thus studies that analyzed tooth loss or number of teeth as a component of oral function and oral health were included. Given that tooth loss is an important epidemiological measure that measures dental status, oral health and function, it should have a more objective and universal quantification [53]. Further, it may be beneficial to formulate and use a gold standard measure of tooth loss that assesses both missing tooth position (qualitative data) and missing teeth number (quantitative data) [54]. Adding to this, many studies had measures of tooth loss along with FTUs (Functional Teeth Units that consider prosthetic contacts) and prosthetic rehabilitation, making it difficult to derive a conclusive result on the association of tooth loss alone with nutritional status. A recent systematic review concluded that nutritional counselling was essential to improve the nutrient intake and prosthetic rehabilitations alone were not sufficient. However, the effect of prosthetic rehabilitation on nutritional intake was beyond the scope of the current overview [55].

Similar heterogeneity was observed in the measurement of nutritional status. In the current overview, malnutrition was considered in the outcome measure. Malnutrition comprises three broad conditions namely, undernutrition, micronutrient-related malnutrition and overweight, obesity and diet-related noncommunicable diseases [56]. Only a few of the included studies analyzed these parameters. Lancker et al. found no significant association between dental condition and serum albumin levels [28]. Gaewkhiew P found that although the most common association was that for weight changes, the findings were inconsistent [25]. They further reported a consistent association of tooth loss with small reductions in dietary cholesterol [25]. Although association of tooth loss to being obese and underweight has been reported, none of the included studies analyzed these parameters [57–59].

Further, nutritional status was predominantly measured by questionnaire-based assessments, followed by anthropometric methods and blood biomarkers. The observed heterogeneity in both the exposure and outcome measure poses a challenge to come to an effective pooled analysis and interpretation of the results.

The current overview provides an extensive quality assessment of the included SRs throwing light on the methodological flaws that may have arisen due to poor conduct of the review. According to the AMSTAR 2 tool assessments done, the key factors affecting the quality were mainly in relation to item 2 (prior registration of the protocols) and item 4 (adequacy of literature search). To increase transparency and improve methodological quality of SRs/MAs a prior registration of the protocol is essential [60]. These findings of quality assessment are similar to those of Pauletto and others, who found less than 1% of recently published SRs in dentistry had high methodological quality [61]. Overall, results in the current overview reveal high heterogeneity in methodology and assessment parameters both at primary and secondary level of studies impairing generalizability of the results.

### Implications for clinical practice and future research

With the established association of tooth loss and nutritional status, clinicians must employ preventative techniques to avert tooth loss, and patients with missing teeth must be examined for nutritional status. High-quality studies with the preferred study design of longitudinal cohorts using longer durations of follow-up must be undertaken. Confounding factors such as sociodemographic characteristics of the population including age, area or country, ethnicity or culture and socioeconomic status and medical conditions must be considered and adjusted. Currently, there is insufficient evidence to include loss of teeth as a risk factor as most of the studies are cross-sectional in design, in which causality cannot be established [62]. Thus, it can be said that even though both the number, location and distribution of the remaining teeth affect the chewing ability of an individual, evidence on correlating these factors to nutritional status is limited and future studies can be undertaken on this [63]. The findings of the study can be utilized to direct efforts to educate the major stakeholders such as patients, physicians and oral health care workers on the impact of tooth loss on nutritional status.

### Strength and limitations

The current study has certain strengths and limitations. A robust methodology was employed to conduct the overview, report the overlap of studies, and quality of both primary and secondary studies. The reporting of the overview has been done as per the modified version of PRIOR checklist [32]. (Supplementary Table 6) The findings of this overview have limitations and therefore need to be interpreted with caution. The review authors were not contacted to clarify the “not reported” or “unclear” contents, as it could have changed the potential underreporting in some domains; the overview methodology may have resulted in the exclusion of some relevant primary studies if they were not included in SRs. As majority of studies were limited to elderly population, the results cannot be generalized to populations of all ages. Lastly, time period of study inclusion was restricted to publications in English and after 2010, which may have resulted in elimination of SRs published prior or in other languages and thus chances of incorporation of publication bias cannot be neglected.

## Conclusion

The currently available evidence indicates that loss of teeth is associated with nutritional status in adults. Individuals with partial or complete edentulism are more likely to be malnourished or at risk of being malnourished. Individuals with poor nutritional status have fewer teeth compared to well-nourished individuals, although clinical implications of this finding are uncertain. Critical analysis of the systematic reviews indicates lack of standardization with considerable methodological variations and heterogeneity in the evidence. Further, quality assessment of the studies reveals low confidence in the available evidence. Future primary studies should be undertaken with a standardized methodology and assessment tools.

### Electronic supplementary material

Below is the link to the electronic supplementary material.

Supplementary Material 1

Supplementary Material 2

Supplementary Material 3

Supplementary Material 4

Supplementary Material 5

Supplementary Material 6

## Data Availability

Data is provided within the manuscript and the supplementary information files.

## Abbreviations

SR/MAs: Systematic Reviews/Meta analysis
PRIOR: A modified version of the Preferred Reporting Items for Overviews of Reviews
DOSS: Dentistry and Oral Sciences Source
NS: Not stated
AMSTAR 2: A Measurement Tool to Assess Systematic Reviews 2
FTUs: Functional Tooth Units
RoB: Risk of Bias
AHRQ: Agency for Healthcare Research and Quality
NOS: Newcastle-Ottawa Scale
MNA: Mini Nutritional Assessment
PK: First author
PK2: Second author
AB: Third author
RKS: Fourth author
VB: Fifth author
