# Supplementary material for: Association of tooth loss and nutritional status in adults: an overview of systematic reviews
Source: BMC Oral Health. 2024 Jul 24;24:838. doi: 10.1186/s12903-024-04602-1 (PMC11267674; doi:10.1186/s12903-024-04602-1)
Supplement: Supplementary file 1 — Supplementary Material 1 [file 12903_2024_4602_MOESM1_ESM.docx]

**Table 1: Details of the databases/online sources searched along with the search strategies.**

| **Sr. No** | **Name of database/online source** | **Search Strategy used** | **Number of articles** | **Date of the Search** |
| --- | --- | --- | --- | --- |
| 1. | PubMed | Nutritional Status [MeSH] OR nutrition* status [tiab] OR malnutrition [tiab] OR "Nutrition Assessment" [MeSH] OR nutrition* assessment [tiab] OR subjective global assessment [tiab] OR mini-nutritional assessment [tiab] OR malnutrition screening tool [tiab] OR malnutrition universal screening tool [tiab]) AND ("Tooth Loss" [MeSH] OR tooth loss [tiab] OR dentate [tiab] OR dentition [tiab] OR edentul*[tiab] OR "Oral Health"[MeSH] | 1153 | 22^nd^ March 2022 |
| 2. | MEDLINE Complete; Health Business Elite; Dentistry & Oral Sciences Source  Interface: Ebscohost. | Tooth loss AND missing teeth AND nutrition or diet or food or nourishment or food intake or eating | 38 | 22^nd^ March 2022 |
| 3. | Cochrane Database | Tooth loss in All Text OR "missing teeth" in Title Abstract Keyword AND nutritional intake in Title Abstract Keyword AND nutrition in Title Abstract Keyword | 304 | 23^rd^ March 2022 |
| 4. | Scopus | Tooth loss AND nutritional assessment AND systematic reviews  Edentulism AND nutritional status AND systematic review | 11 | 23^rd^ March 2022 |
| 5. | Epistemonikos.org | (title:(loss of teeth) OR abstract:(loss of teeth) OR (title:(missing teeth) OR abstract:(missing teeth) AND (title:(nutritional status) OR abstract:(nutritional status)) AND (title:(nutrition) OR abstract:(nutrition)  (title:(loss of teeth OR missing teeth OR edentulism OR absence of teeth AND nutritional status OR nutritional intake OR nutrition) OR abstract:(loss of teeth OR missing teeth OR edentulism OR absence of teeth AND nutritional status OR nutritional intake OR nutrition) | 19 | 23^rd^ March 2022 |
