# Supplementary material for: Association of tooth loss and nutritional status in adults: an overview of systematic reviews
Source: BMC Oral Health. 2024 Jul 24;24:838. doi: 10.1186/s12903-024-04602-1 (PMC11267674; doi:10.1186/s12903-024-04602-1)
Supplement: Supplementary file 2 — Supplementary Material 2 [file 12903_2024_4602_MOESM2_ESM.docx]

**Table 2: Different measures of tooth loss used in the primary studies of the included SR/MAs.**

| **Sr. No** | **Authors** | **Measures to assess Tooth Loss** | **Number of primary studies in the SR that have used the measure** |
| --- | --- | --- | --- |
|  | Algra Yet al. ^24^ | Functional Units | 1 |
|  |  | Number of teeth | 2 |
|  |  | Number of Functional Tooth Units | 2 |
|  |  | Edentulism | 1 |
|  |  | DMFT | 1 |
| 2. | Gaewkhiew P et al. ^25^ | Tooth Loss | 2 |
|  |  | Functional Units | 2 |
|  |  | Chewing Surfaces | 1 |
|  |  | Combination of teeth present and occlusal support | 1 |
|  |  | Edentulism | 1 |
|  |  | Need for dental prosthesis | 2 |
|  |  | Self-reported edentulism | 2 |
|  |  | Self-reported number of teeth lost | 3 |
|  |  | Self-reported Chewing ability | 2 |
| 3. | Hussein S. et al. ^26^ | Number of teeth present | 8 |
|  |  | Number of teeth lost | 1 |
|  |  | Number of occluding pairs | 5 |
|  |  | Functional occlusion | 2 |
|  |  | Edentulous patients with and without complete dentures | 13 |
|  |  | Removable versus fixed prosthesis | 2 |
|  |  | Presence of dental implants | 1 |
| 4. | Lancker V A. et al.^28^ | Number of natural teeth, edentulism, denture use | 7 |
| 5. | Tada A, Miura H. ^16^ | Number of teeth | 9 |
|  |  | Occlusion Pairs | 4 |
|  |  | Dentition status | 5 |
|  |  | Dentition Adequacy | 2 |
|  |  | Denture Status | 7 |
| 6. | Toniazzo M.P et al. ^13^ | Remaining teeth | 19 |
|  |  | Use of dental prosthesis | 16 |
|  |  | Edentulous individuals | 12 |
|  |  | Edentulous wearing dental prosthesis | 2 |
|  |  | Functional Teeth Units or Occluding Pairs | 11 |
|  |  | DMFT | 5 |
| 7. | Zelig R et al. ^17^ | Total edentulism versus remaining teeth | 4 |
|  |  | Number of remaining teeth | 3 |
|  |  | Natural dentition with adequate function versus Functionally inadequate occlusion and no dentures. | 1 |

DMFT=Decayed, missing, filled teeth.
