# Supplementary material for: Association of tooth loss and nutritional status in adults: an overview of systematic reviews
Source: BMC Oral Health. 2024 Jul 24;24:838. doi: 10.1186/s12903-024-04602-1 (PMC11267674; doi:10.1186/s12903-024-04602-1)
Supplement: Supplementary file 3 — Supplementary Material 3 [file 12903_2024_4602_MOESM3_ESM.docx]

**Table 3: Different measures of nutritional status assessment used in the primary studies of the included SR/MAs.**

| **Sr. No.** | **Authors Name** | **Measures of Nutritional Status Assessment** | **Number of primary studies in the SR that have used the measure.** |
| --- | --- | --- | --- |
| 1. | Algra Y et al. ^24^ | MNA | 3 |
|  |  | SGA | 1 |
|  |  | Sarcopenia, MNA-SF, BMI, EAT-10 | 1 |
| 2. | Gaewkhiew P et al. ^25^ | Dietary Assessment: FFQ | 3 |
|  |  | Anthropometric analysis |  |
|  |  | Dietary Recalls | 1 |
|  |  | Number of Items eaten | 1 |
|  |  | Visual Estimation of Plate Waste | 1 |
|  |  | Nutritional Status  Anthropometric analysis | 3 |
|  |  | Waste circumference | 1 |
|  |  | BMI | 1 |
|  |  | Dietary and Nutritional Status: MNA | 1 |
| 3. | Hussein S et al. ^26^ | MNA or MNA- SF | 20 |
| 4. | Lancker V. A et al. ^28^ | MNA | 3 |
|  |  | BMI | 4 |
|  |  | Serum Albumin | 4 |
| 5. | Tada A., Miura H. ^16^ | 24 hour dietary diary | 2 |
|  |  | 24 hour dietary recall | 12 |
|  |  | Frequency of Food intake | 8 |
| 6. | Toniazzo M.P et al. ^13^ | MNA | 18 |
|  |  | MNA-SF | 8 |
| 7. | Zelig R et al. ^17^ | MNA | 3 |
|  |  | MNA-SF | 3 |
|  |  | Self-MNA | 1 |

­­­­­­­

MNA=Mini nutritional Assessment, MNA-SF =Mini Nutritional Assessment Short Form, BMI= Basic Metabolic Index, FFQ= Food Frequency Questionnaire, SGA= Subjective Global Assessment, EAT 10= Eating Assessment Tool
