# Supplementary material for: Association of tooth loss and nutritional status in adults: an overview of systematic reviews
Source: BMC Oral Health. 2024 Jul 24;24:838. doi: 10.1186/s12903-024-04602-1 (PMC11267674; doi:10.1186/s12903-024-04602-1)
Supplement: Supplementary file 4 — Supplementary Material 4 [file 12903_2024_4602_MOESM4_ESM.docx]

**Table 4: Citation Matrix and calculation of the Corrected Covered Area.**

| **Sr. No.** | **Name of Article** | **Algra Y et  al.^24^** | **Gaewkhiew P. et al.^25^** | **Hussein S. et al.^26^** | **Lancker V.A et al.^28^** | **Tada A., Miura H.^16^** | **Toniazzo P. M.  et al. ^13^** |  | **Zelig G. et al. ^17^** |
| --- | --- | --- | --- | --- | --- | --- | --- | --- | --- |
|  | Adiatman M, Ueno M, Ohnuki M, Hakuta C, Shinada K, Kawaguchi Y. Functional tooth units and nutritional status of older people in care homes in Indonesia. Gerodontology. 2013;30: 262-269 |  |  |  |  |  | Yes |  |  |
|  | Akpata, E., Otoh, E., Enwonwu, C., Adeleke, O., & Joshipura, K. Tooth loss, chewing habits, and food choices among older Nigerians in Plateau State: A preliminary study. Community Dentistry and Oral Epidemiology. 2011;39, 409–415. |  |  |  |  | Yes |  |  |  |
|  | Andersson P, Westergren A, Karlsson S, Rahm Hallberg I, Renvert S. Oral health and nutritional status in a group of geriatric rehabilitation patients. Scand J Caring Sci. 2002 Sep;16(3):311-8. |  |  |  | Yes |  |  |  |  |
|  | Andersson, P.; Hallberg, I.; Lorefält, B.; Unosson, M.; Renvert, S. Oral health problems in elderly rehabilitation patients. Int. J. Dent. Hyg. 2004, 2, 70–77. | Yes |  |  | Yes |  |  |  |  |
|  | Appollonio, I., Carabellese, C., Frattola, A., & Trabucchi, M. Influence of dentalstatus on dietary intake and survival in community-dwelling elderly subjects. Age and Ageing. 2007;26, 445–456. |  |  |  |  | Yes |  |  |  |
|  | Awad, M. A., Morais, J. A., Wollin, S., Khalil, A., Gray-Donald, K., & Feine, J. S. Implant overdentures and nutrition: A randomized controlled trial. Journal of Dental Research. 2012;91, 39–46. |  |  |  |  | Yes |  |  |  |
|  | Barrios R, Tsakos G, García-Medina B, Martínez-Lara I, Bravo M. Oral health-related quality of life and malnutrition in patients treated for oral cancer. Support Care Cancer. 2014;22: 2927-2933. |  |  |  |  |  | Yes |  |  |
|  | Blaum CS, Fries BE, Fiatarone MA. Factors associated with low body mass index and weight loss in nursing home residents. J Gerontol A Biol Sci Med Sci. 1995 May;50(3):M162-8. |  |  |  | Yes |  |  |  |  |
|  | Boulos C, Salameh P, Barberger-Gateau P. Factors associated with poor nutritional status among community dwelling Lebanese elderly subjects living in rural areas: results of the AMEL study. |  |  | Yes |  |  |  |  |  |
|  | Bartlett, D. W., Maggio, B., Targett, D., Fenlon, M. R., & Thomas, J. A preliminary investigation into the use of denture adhesives combined with dietary advice to improve diets in complete denture wearers. Journal of Dentistry.2013; 41: 143–147. |  |  |  |  | Yes |  |  |  |
|  | Brennan, D. S., Singh, K. A., Liu, P., & Spencer, A. Fruit and vegetable consumption among older adults by tooth loss and socio-economic status. Australian Dental Journal. 2010;55: 143–149. |  |  |  |  | Yes |  |  |  |
|  | Bradbury, J., Thomason, J. M., Jepson, N. J., Walls, A. W., Mulvaney, C. E., Allen, P. F., et al. Perceived chewing ability and intake of fruit and vegetables. Journal of Dental Research, 87, 720–725 (Erratum in: Journal of Dental Research. 2008;87, 888. |  |  |  |  | Yes |  |  |  |
|  | Bradbury, J., Thomason, J. M., Jepson, N. J., Walls, A. W., Allen, P. F., & Moynihan, P. J. Nutrition counseling increases fruit and vegetable intake in the edentulous. Journal of Dental Research,.2006;85, 463–468. |  |  |  |  | Yes |  |  |  |
|  | Brodeur, J. M., Laurin, D., Vallee, R., & Lachapelle, D. Nutrient intake and gastrointestinal disorders related to masticatory performance in the edentulous elderly. Journal of Prosthetic Dentistry. 1993:70: 468–473. |  |  |  |  | Yes |  |  |  |
|  | Burks CE, Jones CW, Braz VA, Swor RA, Richmond NL, Hwang KS, Hollowell AG, Weaver MA, Platts-Mills TF. Risk Factors for Malnutrition among Older Adults in the Emergency Department: A Multicenter Study. Am J Geriatr Soc 2017;65:1471-7. |  |  | Yes |  |  |  |  |  |
|  | Chai J, Chu FC, Chow TW, Shum NC, Hui WW. Influence of dental status on nutritional status of geriatric patients in a convalescent and rehabilitation hospital. Int J Prosthodont. 2006 May-Jun;19(3):244-9. |  |  |  | Yes |  |  |  |  |
|  | Chen CC, Bai YY, Huang GH, Tang ST. Revisiting the concept of malnutrition in older people. J Clin Nurs. 2007 Nov;16(11). |  |  | Yes |  |  |  |  |  |
|  | Chen CC, Tang ST, Wang C, Huang GH. Trajectory and determinants of nutritional health in older patients during and six-month post-hospitalisation. J Clin Nurs. 2009 Dec;18(23):3299-307. |  |  | Yes |  |  |  |  |  |
|  | Cousson PY, Bessadet M, Nicolas E, Veyrune JL, Lesourd B, Lassauzay C. Nutritional status, dietary intake and oral quality of life in elderly complete denture wearers. Gerodontology. 2012;29: e685-692 |  |  |  |  |  | Yes |  |  |
|  | de Andrade FB, Lebrão ML, de Oliveira Duarte YA, Santos JL. Oral health and changes in weight and waist circumference among community-dwelling older adults in Brazil. J Am Dent Assoc. 2014 Jul;145(7):731-6. |  | Yes |  |  | Yes |  |  |  |
|  | De Marchi RJ, Hugo FN, Hilgert JB, Padilha DM. Association between oral health status and nutritional status in south Brazilian independent-living older people. Nutrition. 2008;24: 546-553. |  |  |  |  | Yes | Yes |  |  |
|  | Dewake N. Relationships amongs sense of coherence, oral health status, nutritional status and care need levels of older adults, according to path analysis. Int Gerontol Geriatr. 2017; 17:88–2083. |  |  | Yes |  |  |  |  |  |
|  | Dion N, Cotart JL, Rabilloud M. Correction of nutrition test errors for more accurate quantification of the link between dental health and malnutrition. Nutrition. 2007;23: 301-307 |  |  |  | Yes |  | Yes |  |  |
|  | El Hélou M, Boulos C, Adib S, Tabbal N. Relationship between oral health and nutritional status in the elderly: A pilot study in Lebanon. Journal of Clinical Gerontology and Geriatrics. 2014;5: 91-95 |  |  |  |  |  | Yes |  |  |
|  | El Osta 2013-El Osta, N.; Hennequin, M.; Tubert-Jeannin, S.; Naaman, N.B.A.; El Osta, L.; Geahchan, N. The pertinence of oral health indicators in nutritional studies in the elderly. Clin. Nutr. 2014, 33, 316–321. [CrossRef] | Yes |  |  |  |  | Yes |  |  |
|  | Enny E, Abdul M, Ruhaya H, Md. Zulkarnain S. Oral hygiene care and nutritional status among institutionalised elderly in Kedah and Kelantan, Malaysia. Malaysian Journal of Nutrition. 2015: 207-217 |  |  |  |  |  | Yes |  |  |
|  | Ervin, R. B., & Dye, B. A.The effect of functional dentition on Healthy Eating Index scores and nutrient intakes in a nationally representative sample of older adults. Journal of Public Health Dentistry. 2009;69, 207–216. |  |  |  |  | Yes |  |  |  |
|  | Ernest, S. L. Dietary intake, food preferences, stimulated salivary flow rate, and masticatory ability in older adults with complete dentitions. Special Care in Dentistry. 1993; 13:102–106. |  |  |  |  | Yes |  |  |  |
|  | Fontijn-Tekamp, F. A., van’t Hof, M. A., Slagter, A. P., & van Waas, M. A. The state of dentition in relation to nutrition in elderly Europeans in the SENECA Study of 1993. European Journal of Clinical Nutrition.1996; 50(Suppl 2), S117–S122. |  |  |  |  | Yes |  |  |  |
|  | Forcanu- Sanjuan. Nutritional screening in case management program for community living older individuals at high risk of hospital administration. Eur Geria Med 2019;9:691-6 |  |  | Yes |  |  |  |  |  |
|  | Feldblum I, German L, Castel H, et al. Characteristics of undernourished older medical patients and the identification of predictors for undernutrition status. Nutr J. 2007;6:37. |  |  | Yes |  |  |  |  |  |
|  | Furuta et al 2018-Furuta M, Takeuchi K, Adachi M, Kinoshita T, Eshima N, Akifusa S, Kikutani T, Yamashita Y. Tooth loss, swallowing dysfunction and mortality in Japanese older adults receiving home care services. Geriatr Gerontol Int. 2018;18(6):873–880. |  |  |  |  |  |  |  | Yes |
|  | Furuta M, Komiya-Nonaka M, Akifusa S, et al. Interrelationship of oral health status, swallowing function, nutritional status, and cognitive ability with activities of daily living in Japanese elderly people receiving home care services due to physical disabilities. Community Dent Oral Epidemiol. 2013;41: 173-181. |  |  |  |  |  | Yes |  |  |
|  | Gil-Montoya JA, Ponce G, Sánchez Lara I, Barrios R, Llodra JC, Bravo M. Association of the oral health impact profile with malnutrition risk in Spanish elders. Arch Gerontol Geriatr. 2013;57: 398-402 |  |  | Yes |  |  | Yes |  |  |
|  | Gil-Montoya JA, Subira C, Ramon JM, Gonzalez- Moles MA. Oral health–related quality of life and nutritional status. J Public Health Dent. 2008; 68(2):88–93. |  |  | Yes |  |  | Yes |  | Yes |
|  | Griep MI, Mets TF, Collys K, Ponjaert-Kristoffersen I, Massart DL. Risk of malnutrition in retirement homes elderly persons measured by the "mininutritional assessment". J Gerontol A Biol Sci Med Sci. 2000;55: M57-63. |  |  |  | Yes |  | Yes |  |  |
|  | Greksa, L. P., Parraga, I. M., & Clark, C. A. (1995). The dietary adequacy of edentulous older adults. Journal of Prosthetic Dentistry.1995; 73, 142–145. |  |  |  |  | Yes |  |  |  |
|  | Group. SGOHR. Oral health issues of Spanish adults aged 65 and over. The Spanish Geriatric Oral Health Research Group. Int Dent J. 2001;51: 228- 234. |  |  |  |  |  | Yes |  |  |
|  | Gunji, A., Kimoto, S., Koide, H., Murakami, H., Matsumaru, Y., Kimoto, K., et al. Investigation on how renewal of complete dentures impact on dietary and nutrient adequacy in edentulous patients. Journal of Prosthodontic Research. 2009;53, 180–184. |  |  |  |  | Yes |  |  |  |
|  | Holst.Yifter E Lindgren,Surowiak M. Nutritional screening and risk factors in elderly hospitalized patients: association to clinical outcome? Scan J Caring 2013;27:953-61 |  |  | Yes |  |  |  |  |  |
|  | Horn VJ, Hodge WC, Treuer JP. Dental condition and weight loss in institutionalized demented patients. Spec Care Dentist. 1994 May-Jun;14(3):108-11. |  |  |  | Yes |  |  |  |  |
|  | H.C. Hung, G. Colditz, K.J. Joshipura. The association between tooth loss and the self-reported intake of selected CVD-related nutrients and foods among US women. Community Dent Oral Epidemiol. 2005;33 :167-73. |  | Yes |  |  | Yes |  |  |  |
|  | Huppertz 2017-Huppertz, V.A.; van der Putten, G.-J.; Halfens, R.J.; Schols, J.M.; de Groot, L. Association between Malnutrition and Oral Health in Dutch Nursing Home Residents: Results of the LPZ Study. J. Am. Med. Dir. Assoc. 2017, 18, 948–954. [CrossRef] | Yes |  |  |  |  |  |  |  |
|  | Iizaka S, Tadaka E, Sanada H. Comprehensive assessment of nutritional status and associated factors in the healthy, community-dwelling elderly. Geriatr Gerontol Int. 2008 Mar;8(1):24-31. |  |  | Yes |  |  |  |  |  |
|  | Hung HC, Willett W, Ascherio A, Rosner BA, Rimm E, Joshipura KJ. Tooth loss and dietary intake. J Am Dent Assoc. 2003 Sep;134(9):1185-92. |  | Yes |  |  |  |  |  |  |
|  | M. Iwasaki, A. Yoshihara, H. Ogawa, M. Sato, K. Muramatsu, R. Watanabe, et al. Longitudinal association of dentition status with dietary intake in Japanese adults aged 75 to 80 years. J Oral Rehabil. 2016;43 : 737-44. |  | Yes |  |  |  |  |  |  |
|  | K.J. Joshipura, W.C. Willett, C.W. Douglass. The impact of edentulousness on food and nutrient intake. J Am Dent Assoc. 1996; 127:459-67. |  | Yes |  |  | Yes |  |  |  |
|  | Jürschik P, Torres J, Solá R, Nuin C, Botigué T, Lavedán A. High rates of malnutrition in older adults receiving different levels of health care in Lleida, Catalonia: an assessment of contributory factors. J Nutr Elder. 2010 Oct;29(4):410-22. |  |  | Yes |  |  |  |  |  |
|  | Kagawa, R., Ikebe, K., Inomata, C., Okada, T., Takeshita, H., Kurushima, Y., et al. Effect of dental status and masticatory ability on decreased frequency of fruit and vegetable intake in elderly Japanese subjects. International Journal of Prosthodontics. 2012;25:368–375. |  |  |  |  | Yes |  |  |  |
|  | Kiesswetter, E.; Hengeveld, L.M.; Keijser, B.J.; Volkert, D.; Visser, M. Oral health determinants of incident malnutrition in community-dwelling older adults. J. Dent. 2019;85:73–80. | Yes |  |  |  |  |  |  |  |
|  | Kikutani T, Yoshida M, Enoki H, Yamashita Y, Akifusa S, Shimazaki Y, Hirano H, Tamura F. Relationship between nutritional status and dental occlusion in community-dwelling frail elderly people. Geriatr Gerontol Int. 2013;13(1):50–54 |  |  | Yes |  |  | Yes |  | Yes |
|  | Krall, E., Hayes, C., & Garcia, R. (1998). How dentition status and masticatory function affect nutrient intake. Journal of the American Dental Association 1998; 129:1261–1269. |  |  |  |  | Yes |  |  |  |
|  | Krzyminska-Siemaszko R, Chudek J, Suwalska A, Lewandowicz M, Mossakowska M, Kroll-Balcerzak R, Wizner B, Tobis S, Mehr K, Wieczorowska-Tobis K. 2016. Health status correlates of malnutrition in the polish elderly population—results of the PolSenior study. Eur Rev Med Pharmacol Sci.2016; 20(21):4565–4573. |  |  |  |  |  |  |  | Yes |
|  | Kucuk EO, Kapucu S. Malnutrition in Elderly Staying in Nursing Homes. KONURALP TIP Derg 2017; 9:222–7 |  |  | Yes |  |  |  |  |  |
|  | J. Kwon, T. Suzuki, S. Kumagai, S. Shinkai, H. Yukawa. Risk factors for dietary variety decline among Japanese elderly in a rural community: a 8-year follow-up study from TMIG-LISA. Eur J Clin Nutr. 2006;60 : 305-11. |  | Yes |  |  |  |  |  |  |
|  | Lachapelle, D., Brodeur, J. M., Simard, P. L., Vallee, R., & Moisan, J. Masticatory ability and dietary adequacy of elderly denture wearers. Journal of the Canadian Dental Association. 1992;53, 145–150. |  |  |  |  | Yes |  |  |  |
|  | Lamy M, Mojon P, Kalykakis G. Oral status and nutrition in the institutionalized elderly. J Dent 1999; 27:443–8. |  |  | Yes | Yes |  | Yes |  |  |
|  | Laurin, D., Brodeur, J. M., Bourdages, J., Valle ́ e, R., & Lachapelle, D. Fibre intake in elderly individuals with poor masticatory performance. Journal of the Canadian Dental Association. 1994;60, 443–446 |  |  |  |  | Yes |  |  |  |
|  | J.S. Lee, R.J. Weyant, P. Corby, S.B. Kritchevsky, T.B. Harris, R. Rooks, et al. Edentulism and nutritional status in a biracial sample of well-functioning, community-dwelling elderly: the health, aging, and body composition study. Am J Clin Nutr. 2004;79:295-302. |  | Yes |  |  | Yes |  |  |  |
|  | Liedberg, B., Stoltze, K., Norle ́n, P., & Owall, B. ‘Inadequate’ dietary habits and mastication in elderly men. Gerodontology.2007; 24, 41–46. |  |  |  |  | Yes |  |  |  |
|  | Lin, Y. C., Chen, J. H., Lee, H. E., Yang, N. P., & Chou, T. M.. The association of chewing ability and diet in elderly complete denture patients. International Journal of Prosthodontics. 2010;23:127–128. |  |  |  |  | Yes |  |  |  |
|  | Lindmark 2017-Lindmark, U.; Jansson, H.; Lannering, C.; Johansson, L. Oral health matters for the nutritional status of older persons-A population-based study. J. Clin. Nurs. 2018, 27, 1143–1152. | Yes |  | Yes |  |  |  |  |  |
|  | Lopez-Jornet P, Saura-Perez M, Llevat-Espinosa N. Effect of oral health dental state and risk of malnutrition in elderly people. Geriatr Gerontol Int 2013;13:43–9. |  |  | Yes |  |  | Yes |  | Yes |
|  | Marshall, T. A., Warren, J. J., Hand, J. S., Xie, X. J., & Stumbo, P. J. Oral health, nutrient intake and dietary quality in the very old. Journal of the American Dental Association. 2002;133:1369–1379. |  |  |  |  | Yes |  |  |  |
|  | Mesas, A.E.; De Andrade, S.; Cabrera, M.A.S.; Bueno, V.L.R.D.C. Salud oral y déficit nutricional en adultos mayores no institucionalizados en Londrina, Paraná, Rev Bras Epidemiol. 2010;13: 434-445 | Yes |  |  |  |  | Yes |  |  |
|  | Mojon P, Budtz-Jørgensen E, Rapin CH. Relationship between oral health and nutrition in very old people. Age Ageing. 1999 Sep;28(5):463-8. |  |  |  | Yes |  |  |  |  |
|  | Morais, J. A., Heydecke, G., Pawliuk, J., Lund, J. P., & Feine, J. S. The effects of mandibular two-implant overdentures on nutrition in elderly edentulous individ-uals. Journal of Dental Research. 2003;82: 53–58. |  |  |  |  | Yes |  |  |  |
|  | Moynihan, P. J., Butler, T. J., Thomason, J. M., & Jepson, N. J. Nutrient intake in partially dentate patients: The effect of prosthetic rehabilitation. Journal of Den-tistry.2000; 28:557–563. |  |  |  |  | Yes |  |  |  |
|  | Mudge AM, Ross LJ, Young AM. Helping understand nutritional gaps in the elderly (HUNGER): A prospective study of patient factors associated with inadequate nutritional intake in older medical inpatients. Clin Nutr 2011; 30:320–5. https://doi.org/10.1016/j.clnu.2010.12.007 |  | Yes | Yes |  |  |  |  |  |
|  | Nordenram G, Ljunggren G, Cederholm T. Nutritional status and chewing capacity in nursing home residents. Aging (Milano). 2001 Oct;13(5):370-7. |  |  |  | Yes |  |  |  |  |
|  | Nykänen I, Lönnroos E, Kautiainen H. Nutritional screening in a population- based cohort of community-dwelling older people. Eur J Public Health 2013; 23:405–9. https://doi.org/10.1093/eurpub/cks026 |  |  | Yes |  |  |  |  |  |
|  | Okabe Y, Furuta M, Akifusa S, et al. Swallowing Function and Nutritional Status in Japanese Elderly People Receiving Home-care Services: A 1-year Longitudinal Study. J Nutr Health Aging. 2016;20: 697-704 |  |  |  |  |  | Yes |  |  |
|  | Okada K, Enoki H, Izawa S, Iguchi A, Kuzuya M. Association between masticatory performance and anthropometric measurements and nutritional status in the elderly. Geriatr Gerontol Int. 2010 Jan;10(1):56-63. |  |  |  | Yes |  |  |  |  |
|  | Osterberg, T., Tsuga, K., Rothenberg, E., Carlsson, G. E., & Steen, B. Masticatory ability in 80-year-old subjects and its relation to intake of energy, nutrients and food items. Gerodontology.2002; 19: 95–101. |  |  |  |  | Yes |  |  |  |
|  | Pillai RS, Mathur VP, Jain V, et al. Association between dental prosthesis need, nutritional status and quality of life of elderly subjects. Qual Life Res. 2015;24: 2863-2871 |  |  |  |  |  | Yes |  |  |
|  | Paillaud E, Merlier I, Dupeyron C, Scherman E, Poupon J, Bories PN. Oral candidiasis and nutritional deficiencies in elderly hospitalised patients. Br J Nutr. 2004 Nov;92(5):861-7. |  |  |  | Yes |  |  |  |  |
|  | Poisson P, Laffond T, Campos S. Relationships between oral health, dysphagia and undernutrition in hospitalised elderly patients. Gerodontology 2016; 33:161–168. https://doi.org/10.1111/ger.12123 | Yes |  | Yes |  |  | Yes |  |  |
|  | Rauen MS, Moreira EA, Calvo MC, Lobo AS. Oral condition and its relationship to nutritional status in the institutionalized elderly population. J Am Diet Assoc. 2006 Jul;106(7):1112-4. |  |  |  | Yes |  |  |  |  |
|  | Ritchie C.S, K. Joshipura, R.A. Silliman, B. Miller, C.W. Douglas. Oral health problems and significant weight loss among community-dwelling older adults. J Gerontol A Biol Sci Med Sci.2000; 55:366-71. |  | Yes |  |  |  |  |  |  |
|  | Saarela RKT, Lindroos E, Soini H. Dentition, nutritional status and adequacy of dietary intake among older residents in assisted living facilities. Gerodontology 2016;33:225–32. |  |  | Yes |  |  |  |  | Yes |
|  | Saarela RKT, Soini H, Hiltunen K. Dentition status, malnutrition and mortality among older service housing residents. J Nutr Heal Aging 2014; 18:34–8. https://doi.org/10.1007/s12603-013-0358-3 |  |  | Yes |  |  |  |  |  |
|  | Saarela RKT, Savikko NM, Soini H, Muurinen S, Suominen MH, Kautiainen H, Pitkala KH. Burden of Oral Symptoms and Health-Related Quality of Life in Long-Term Care Settings in Helsinki, Finland. J Nutr Health Aging. 2019;23(10):1021-1025. |  |  | Yes |  |  |  |  |  |
|  | Saarela RK, Soini H, Muurinen S, Suominen MH, Pitkälä KH. Oral hygiene and associated factors among frail older assisted living residents. Spec Care Dentist. 2013 Mar-Apr;33(2):56-61. |  |  | Yes |  |  |  |  |  |
|  | Sadamori S, Hayashi S, Hamada T. The relationships between oral status, physical and mental health, nutritional status and diet type in elderly Japanese women with dementia. Gerodontology. 2008 Dec;25(4):205-9. |  |  |  | Yes |  |  |  |  |
|  | Samnieng 2011-Samnieng, P.; Ueno, M.; Shinada, K.; Zaitsu, T.; Wright, F.A.C.; Kawaguchi, Y. Oral Health Status and Chewing Ability is Related to Mini-Nutritional Assessment Results in an Older Adult Population in Thailand. J. Nutr. Gerontol. Geriatr. 2011;30:291–304. | Yes |  |  |  |  | Yes |  |  |
|  | N. Sato, T. Ono, H. Kon, N. Sakurai, S. Kohno, A. Yoshihara, et al. Ten-year longitudinal study on the state of dentition and subjective masticatory ability in community-dwelling elderly people. J Prosthodont Res. 2016; 60 : 177-84. |  | Yes |  |  |  |  |  |  |
|  | Sahyoun, N. R., Lin, C. L.,&Krall, E.(2003). Nutritional status ofthe older adultis associated with dentition status. Journal of the American Dietetic Association, 103, 61–66. |  |  |  |  | Yes |  |  |  |
|  | Sahyoun, N. R., & Krall, E. Low dietary quality among older adults with self-perceived ill-fitting dentures. Journal of the American Dietetic Association. 2003;103,1494–1499. |  |  |  |  | Yes |  |  |  |
|  | Sebring, N. G., Guckes, A. D., Li, S. H., & McCarthy, G. R. Nutritional adequacy of reported intake of edentulous subjects treated with new conventional or implant-supported mandibular dentures. Journal of Prosthetic Dentistry. 1995;74, 358–363. |  |  |  |  | Yes |  |  |  |
|  | Sheiham, A., Steele, J. G., Marcenes, W., Lowe, C., Finch, S., Bates, C. J., et al. The relationship among dental status, nutrient intake, and nutritional status in older people. Journal of Dental Research.2001; 80, 408–413. |  |  |  |  | Yes |  |  |  |
|  | Shiraishi A, Yoshimura Y, Wakabayashi H, Tsuji Y. Poor oral status is associated with rehabilitation outcome in older people. Geriatr Gerontol Int 2017; 17:598–604. https://doi.org/10.1111/ggi.12763 |  |  | Yes |  |  |  |  |  |
|  | Shinkai, R. S., Hatch, J. P., Sakai, S., Mobley, C. C., & Rugh, J. D. Dietary intake inedentulous subjects with good and poor quality complete dentures. Journal of Dental Research. 2002;87:490–498. |  |  |  |  | Yes |  |  |  |
|  | Soini, H.; Muurinen, S.; Routasalo, P.; Sandelin, E.; Savikko, N.; Suominen, M.; Ainamo, A.; Pitkala, K.H. Oral and nutritional status–Is the MNA a useful tool for dental clinics. J. Nutr. Health Aging 2006, 10, 500–501. | Yes |  | Yes | Yes |  |  |  |  |
|  | Soini H, Routasalo P, Lauri S, Ainamo A. Oral and nutritional status in frail elderly. Spec Care Dentist. 2003;23: 209-215. |  |  |  |  |  | Yes |  |  |
|  | Solemdal K, Sandvik L, Møinichen-Berstad C, Skog K, Willumsen T, Mowe M. Association between oral health and body cell mass in hospitalised elderly. Gerodontology. 2012;29: e1038-1044 |  |  | Yes |  |  | Yes |  |  |
|  | Srinivasulu G, Fareed N, Sudhir KM, Krishna Kumar RV. Relationship between stimulated salivary factors, dental caries status and nutritional condition among institutionalized elderly people. Oral Health Dent Manag. 2014;13: 49-53 |  |  |  |  |  | Yes |  |  |
|  | Stoffel LMB, Muniz FWMG, Colussi PRG. Nutritional assessment and associated factors in the elderly: A population-based cross-sectional study. Nutrition 2018; 55–56:104–10. |  |  | Yes |  |  |  |  |  |
|  | Subira C, Ramon JM, Almerich JM. Oral health issues of Spanish adults aged 65 and over. Int Dent J 2001; 51:228–34 |  |  | Yes |  |  |  |  |  |
|  | Sullivan DH, Martin W, Flaxman N, Hagen JE. Oral health problems and involuntary weight loss in a population of frail elderly. J Am Geriatr Soc. 1993 Jul;41(7):725-31. |  |  |  | Yes |  |  |  |  |
|  | Syrjälä AM, Pussinen PI, Komulainen K, et al. Salivary flow rate and risk of malnutrition - a study among dentate, community-dwelling older people. Gerodontology. 2013;30: 270-275 |  |  | Yes |  |  | Yes |  |  |
|  | Tsai AC, Chang TL. Association of dental prosthetic condition with food consumption and risk of malnutiriton and a follow up 4 year mortality risk in elderly Taiwanese. J Nutri Heal Aging 2011;15:265. |  |  | Yes |  | Yes |  |  |  |
|  | Takahashi 2018-Takahashi, M.; Maeda, K.; Wakabayashi, H. Prevalence of sarcopenia and association with oral health-related quality of life and oral health status in older dental clinic outpatients. Geriatr. Gerontol. Int. 2018, 18, 915–921. [ | Yes |  |  |  |  |  |  |  |
|  | Wakabayashi H, Matsushima M, Ichikawa H, Murayama S, Yoshida S, Kaneko M, Mutai R. Occlusal Support, Dysphagia, Malnutrition, and Activities of Daily Living in Aged Individuals Needing Long-Term Care: A Path Analysis. J Nutr Health Aging. 2018;22(1):53-58. |  |  | Yes |  |  |  |  |  |
|  | Wu LL, Cheung KY, Lam PYP, Gao XL. Oral Health Indicators for Risk of Malnutrition in Elders. J Nutr Health Aging. 2018;22(2):254-261. |  |  | Yes |  |  |  |  |  |
|  | Wöstmann B, Michel K, Brinkert B, Melchheier-Weskott A, Rehmann P, Balkenhol M. Influence of denture improvement on the nutritional status and quality of life of geriatric patients. J Dent. 2008;36: 816-821 |  |  |  |  |  | Yes |  |  |
|  | Yoshida, M., Kikutani, T., Yoshikawa, M., Tsuga, K., Kimura, M., & Akagawa, Y. Correlation between dental and nutritional status in community-dwelling elderly Japanese. Geriatrics & Gerontology International. 2011; 11:315–319. |  |  |  |  | Yes |  |  |  |
|  | Zelig R, Byham-Gray L, Singer SR, Hoskin ER, Fleisch Marcus A, Verdino G, Radler DR, Touger-Decker R. 2018. Dentition and malnutrition risk in community dwelling older adults. J Aging Res Clin Pract. 7:107–114 |  |  |  |  |  |  |  | Yes |

N=137 (Total number of studies in the reviews)

r=107, C=7

CCA= N-r/r(c)-r. =137-107/107(7)-107, = 0.04
