# Supplementary material for: Association of tooth loss and nutritional status in adults: an overview of systematic reviews
Source: BMC Oral Health. 2024 Jul 24;24:838. doi: 10.1186/s12903-024-04602-1 (PMC11267674; doi:10.1186/s12903-024-04602-1)
Supplement: Supplementary file 5 — Supplementary Material 5 [file 12903_2024_4602_MOESM5_ESM.docx]

**Table 5: Summary of quality assessment of primary studies done by included SRs.**

| **Sr. No** | **Author** | **Tool used** | **Overall result of quality assessment.** |
| --- | --- | --- | --- |
|  | Algra Y. et al.^24^ | New Ottawa Scale | Clinical heterogeneity was observed along with low level of evidence. |
|  | Gaewkhiew P. et al.^25^ | Newcastle-Ottawa  Quality Assessment  Scale | Only one study qualified as fair, while all others scored poor. |
| 3. | Hussain S. et al.^26^ | Agency for Healthcare Research and Quality and GRADE. | Overall, 72.7% was rated as high risk of bias, 15.2% as unclear and only 12.1% of the studies were rated "Low Risk of Bias" |
| 4. | Lancker V.A et al.^28^ | Modified Version of the Quality Assessment and Vality tool for Correlational Studies. | Only one study was rated as high quality, rest all studies were medium in quality. |
| 5. | Tada A. et al.^16^ | Oxford Center for Evidence based medicine | Overall the quality was highly variable. |
| 6. | Toniazzo M. et al.^13^ | Agency for Healthcare Research and Quality (AHRQ) and Newcastle Ottawa quality assessment scale | High variability between the studies was observed. Overall assessment is unclear. |
| 7. | Zelig R et al.^17^ | Modified New Castle-Ottawa Scale | Overall risk of bias was moderate. |
